# Clinical remission and associated factors in severe eosinophilic asthma patients treated with benralizumab: a real-world study

**DOI:** 10.1186/s12890-026-04518-2

**Published:** 2026-07-28

**Authors:** Melis Yağdıran, Kurtuluş Aksu

**Affiliations:** Division of Immunology and Allergy Diseases, Department of Chest Diseases, University of Health Sciences, Ankara Atatürk Sanatorium Training and Research Hospital, Ankara, Türkiye

**Keywords:** Severe asthma, Benralizumab, Eosinophilic asthma, Clinical remission, Exacerbation, Biologic therapy

## Abstract

**Background:**

This study aimed to evaluate changes in clinical, functional, and inflammatory parameters in patients with severe eosinophilic asthma receiving benralizumab treatment, to determine clinical remission rates, and to explore factors associated with clinical remission.

**Methods:**

This retrospective single-center real-world study included 33 patients with severe eosinophilic asthma treated with benralizumab at a tertiary referral center. Demographic characteristics, clinical findings, pulmonary function test results, peripheral blood eosinophil counts, and treatment responses were obtained from medical records. Baseline findings were compared with those obtained after at least 12 months of treatment. Clinical remission was defined as the absence of exacerbations, no maintenance oral corticosteroid use, well-controlled asthma, and stable or improved lung function. Factors associated with clinical remission were evaluated using multivariable Firth’s penalized logistic regression.

**Results:**

The mean age of the 33 included patients was 54.6 ± 13.8 years, and 60.6% were female. After 12 months of treatment, clinical remission was achieved in 75.8% (25/33) of patients. The proportion of patients with well-controlled asthma increased to 90.9%, while maintenance oral corticosteroid use decreased from 48.5% to 24.2% (*p* = 0.008). The median annual exacerbation rate decreased from 2 to 0 (*p* < 0.001). Mean FEV₁ increased from 1514 mL to 2188 mL (+ 674 mL; *p* < 0.001), and FEV₁ (% predicted) improved from 54.9% to 76.8% (*p* < 0.001). Peripheral blood eosinophil counts decreased from a median of 720 cells/µL to 0 cells/µL (*p* < 0.001). In multivariable analysis, lower baseline exacerbation frequency (OR 0.52, 95% CI 0.27–0.91; *p* = 0.021) and higher baseline FEV₁ (per 100 mL increase; OR 1.27, 95% CI 1.01–1.59; *p* = 0.037) were associated with clinical remission.

**Conclusions:**

Benralizumab was associated with high clinical remission rates, substantial reductions in exacerbations, and significant improvements in lung function in patients with severe eosinophilic asthma. Given the limited sample size, the findings from the exploratory multivariable analysis should be interpreted with caution and require confirmation in larger prospective studies.

## Introduction

 Asthma is a heterogeneous chronic airway disease characterized by persistent inflammation and represents a major cause of morbidity worldwide [1]. Despite current treatment strategies, a considerable proportion of patients remain inadequately controlled, particularly those with severe asthma, who frequently experience recurrent exacerbations, increased healthcare utilization, and impaired quality of life [1, 2]. Repeated exacerbations, long-term oral corticosteroid (OCS) exposure, and progressive airway damage continue to represent important clinical challenges in this patient population.

Advances in the understanding of asthma phenotypes and endotypes have led to the development of targeted biologic therapies. In patients with severe eosinophilic asthma, treatments targeting the interleukin-5 (IL-5) pathway have become an important therapeutic option. Benralizumab is a monoclonal antibody directed against the IL-5 receptor α that induces rapid and near-complete eosinophil depletion through antibody-dependent cell-mediated cytotoxicity. Previous clinical trials have demonstrated that benralizumab significantly reduces exacerbation rates and improves lung function in patients with severe eosinophilic asthma [3, 4].

In recent years, the treatment goals in severe asthma have evolved beyond symptom control, and the concept of “clinical remission” has gained increasing attention. Clinical remission is generally defined as the absence of exacerbations and OCS use, together with well-controlled symptoms and stable lung function over time [5]. Emerging evidence suggests that biologic therapies may enable some patients to achieve these multidimensional treatment targets [6].

Recent long-term real-world studies have further demonstrated the sustained effectiveness of benralizumab and its potential role in achieving clinical remission. Studies with extended follow-up periods have reported substantial reductions in exacerbation rates, improvements in lung function, and increased remission rates among patients receiving benralizumab treatment [7]. However, real-world data evaluating clinical remission after benralizumab therapy remain limited, and predictors of remission have not yet been fully clarified across different patient populations.

The aim of this study was to retrospectively evaluate the demographic characteristics, clinical findings, functional outcomes, and laboratory parameters of patients with severe eosinophilic asthma treated with benralizumab in our clinic, and to determine clinical remission rates and factors associated with remission.

## Methods

### Study design and patients

This was a retrospective study based on routinely collected clinical data. Although the study included patients treated between October 2022 and March 2025, ethical approval was obtained on March 25, 2026, before data retrieval and analysis of the study cohort.

Clinical remission was defined according to current consensus recommendations as the absence of exacerbations, no maintenance oral corticosteroid use, well-controlled asthma (ACT ≥ 20), and stable or improved lung function. In this retrospective study, the lung function component was assessed by comparing FEV₁ measurements obtained at baseline and at the 12-month follow-up visit.

No interventional procedures were performed as part of the study, and only existing clinical records were analyzed. Patients younger than 18 years of age and those with incomplete medical records were excluded.

### Ethical considerations

Ethical approval was obtained from the Scientific Research Ethics Committee of University of Health Sciences Ankara Atatürk Sanatorium Training and Research Hospital (Approval No: 2024-BÇEK/509; March 25, 2026). The study was conducted in accordance with the Declaration of Helsinki. Given the retrospective nature of the study, the requirement for informed consent was waived.

### Statistical analysis

Statistical analyses were performed using IBM SPSS Statistics for Windows (Version 25.0; IBM Corp., Armonk, NY, USA) and R software (Version 4.6.0; R Foundation for Statistical Computing, Vienna, Austria). Continuous variables were expressed as mean ± standard deviation (SD) or median (interquartile range [IQR]) according to data distribution, while categorical variables were presented as frequencies and percentages.

The normality of continuous variables was assessed using the Kolmogorov–Smirnov test. Comparisons between baseline and 12-month follow-up measurements were performed using the paired-samples t-test for normally distributed variables and the Wilcoxon signed-rank test for non-normally distributed variables. Categorical variables were compared using the McNemar test.

Patients were categorized into remission and non-remission groups according to the predefined clinical remission criteria. Comparisons between these groups were performed using the independent samples t-test or Mann–Whitney U test for continuous variables, and the chi-square test or Fisher’s exact test for categorical variables, as appropriate.

To explore factors associated with clinical remission, univariable logistic regression analyses were initially performed. Variables with a p-value < 0.10 in the univariable analyses were subsequently included in a multivariable Firth’s penalized logistic regression model, which was applied to reduce small-sample bias and the potential risk of overfitting associated with conventional maximum likelihood estimation. Results are presented as odds ratios (ORs) with 95% confidence intervals (CIs). Receiver operating characteristic (ROC) curve analysis was performed to evaluate the discriminative ability of the model; however, given the limited sample size and the small number of non-remission events, these findings were considered exploratory and interpreted with caution.

A two-sided p-value < 0.05 was considered statistically significant.

## Results

A total of 33 patients with severe eosinophilic asthma receiving benralizumab were included. The mean age was 54.6 ± 13.8 years, and 60.6% were female. Baseline characteristics are summarized in Table 1.

**Table 1:** Demographic and baseline characteristics

Variable	Value
Number of patients (n)	33
Age (years)	54.6 ± 13.8
Female, n (%)	20 (60.6)
Male, n (%)	13 (39.4)
BMI (kg/m²)	29.3 ± 4.9
Disease duration (years)	20.3 ± 12.4
Current smokers, n (%)	5 (15.2)
Atopy, n (%)	6 (18.2)
Nasal polyps, n (%)	12 (36.4)
Rhinitis, n (%)	5 (15.2)
NSAID hypersensitivity, n (%)	8 (24.2)
OCS use at baseline, n (%)	16 (48.5)
Eosinophils (median, cells/µL)	720 (450–980)

At 12 months, marked improvements were observed across all clinical outcomes. Clinical remission was achieved in 75.8% of patients (25/33), while asthma control (ACT ≥ 20) was reached in 90.9% (30/33). The proportion of patients requiring oral corticosteroids (OCS) decreased significantly from 48.5% to 24.2% (*p* = 0.008). Annual exacerbation rates declined substantially, with the median decreasing from 2 to 0 (*p* < 0.001) (Table 2).

**Table 2:** Treatment outcomes (baseline vs. 12 months)

Parameter	Baseline	12 Months	*p*-value
Exacerbations (median, IQR)	2 (1–3)	0 (0–1)	< 0.001
FEV1 (mL)	1514 ± 722	2188 ± 871	< 0.001
FEV1 (% predicted)	54.9 ± 18.6	76.8 ± 19.4	< 0.001
FEV1/FVC (%)	64.8 ± 10.5	74.5 ± 9.8	< 0.001
Eosinophils (median, cells/µL)	720 (450–980)	0 (0–0)	< 0.001
Asthma control (ACT ≥ 20), n (%)	9 (27.3)	30 (90.9)	< 0.001
OCS use, n (%)	16 (48.5)	8 (24.2)	0.008
Clinical remission, n (%)	–	25 (75.8)	–
ACT score, median (IQR)	13 (10–16)	22 (19–24)	< 0.001

**Fig. 1:**
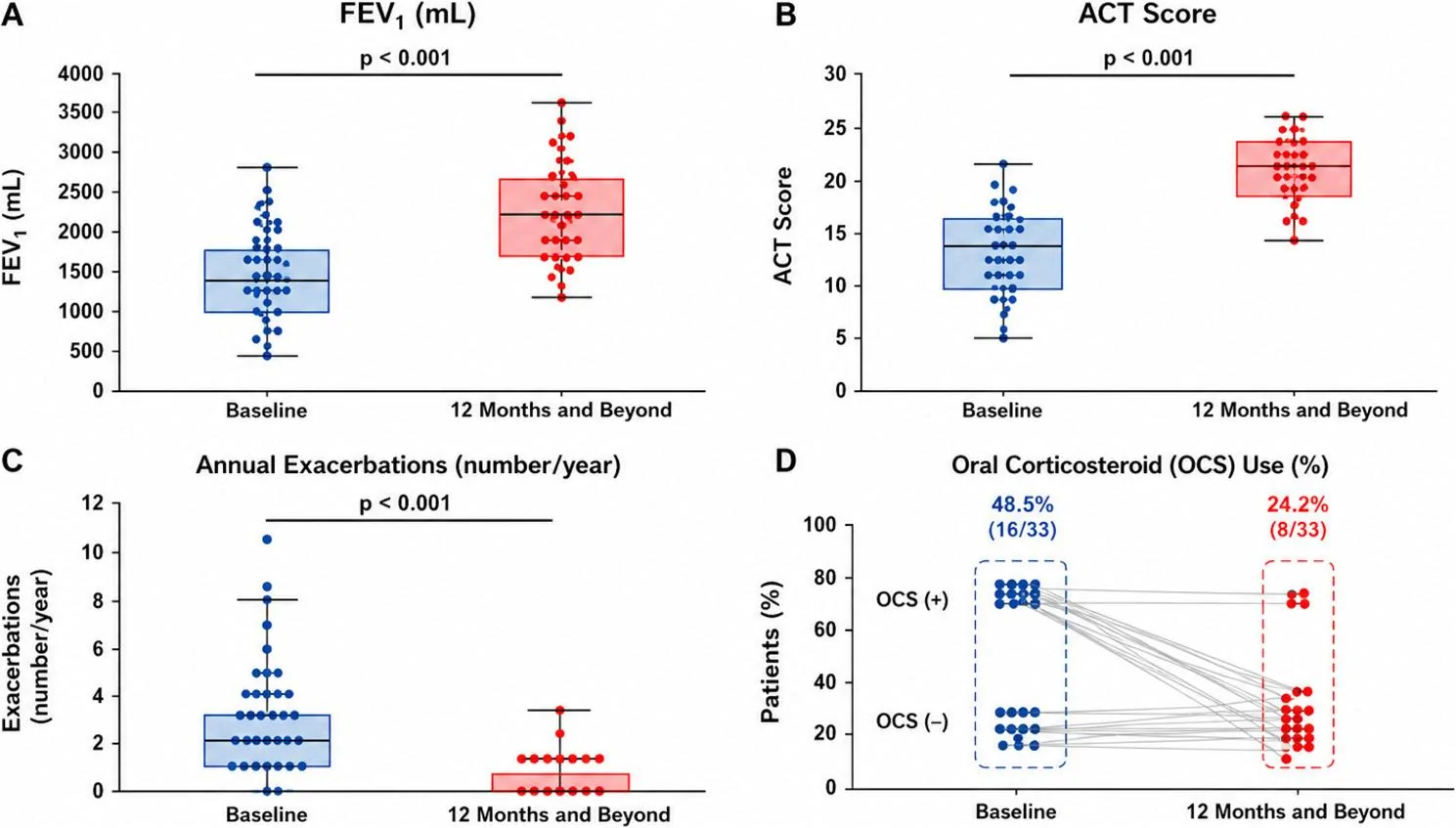
Changes in clinical outcomes after 12 months and beyond of benralizumab therapy in patients with severe asthma. **A** $\mathrm{FEV}_{1}$ (mL) significantly increased. **B** ACT scores significantly improved. **C** Annual exacerbation rates significantly decreased. **D** The proportion of patients using oral corticosteroids (OCS) decreased.Boxes represent interquartile range (IQR) with median line; whiskers indicate $1.5\times\;\mathrm{IRQ}$; dots represent individual patient values. Paired proportions are shown in (**D**) with lines connecting individual patients

Significant improvements were also observed in lung function. Mean FEV1 increased from 1514 mL to 2188 mL (mean difference: +674 mL; *p* < 0.001), and FEV1 (% predicted) improved from 54.9% to 76.8% (*p* < 0.001). ACT scores also increased markedly, reflecting improved symptom control (Fig. 1A–B). Although the median post-treatment eosinophil count was 0 cells/µL (IQR, 0–0), three patients had low but detectable eosinophil counts at follow-up: two patients had 10 cells/µL and one patient had 20 cells/µL. No patient showed persistent eosinophilia (Table 2).

When stratified according to clinical remission status, patients who achieved remission had significantly lower baseline exacerbation rates (*p* = 0.015) and higher baseline FEV_1_ values (*p* = 0.015) than those who did not achieve remission. No significant differences were observed between the two groups regarding age, sex, body mass index, disease duration, baseline maintenance oral corticosteroid use, or the presence of nasal polyps (Table 3).

**Table 3:** Comparison of baseline characteristics according to clinical remission status

Variable	Remission (*n* = 25)	No Remission (*n* = 8)	*p*-value
Age, years	54.1 ± 13.7	56.1 ± 15.0	0.739
Female, n (%)	16 (64.0)	4 (50.0)	0.431
BMI, kg/m²	28.8 ± 5.0	30.7 ± 4.4	0.322
Disease duration, years	22.1 ± 12.2	14.5 ± 12.1	0.147
Nasal polyps, n (%)	9 (36.0)	3 (37.5)	1.000
Baseline OCS use, n (%)	11 (44.0)	5 (62.5)	0.438
Baseline exacerbations	1.6 ± 0.8	2.8 ± 1.0	0.015
Baseline FEV₁ (mL)	1600 ± 450	1250 ± 380	0.015

To further explore factors associated with clinical remission, variables showing a potential association in the univariable analyses were entered into a multivariable Firth’s penalized logistic regression model. Lower baseline exacerbation frequency (OR 0.52, 95% CI 0.27–0.91; *p* = 0.021) and higher baseline FEV1 (per 100 mL increase; OR 1.27, 95% CI 1.01–1.59; *p* = 0.037) were independently associated with achieving clinical remission (Table 4). Given the limited sample size and the small number of non-remission events, these findings should be considered exploratory.

**Table 4:** Multivariable logistic regression for clinical remission

Variable	OR	95% CI	*p*-value
Baseline exacerbation rate	0.52	0.27–0.91	0.021
Baseline FEV1 (per 100 mL increase)	1.27	1.02–1.59	0.037

## Discussion

In the present study, clinical remission was achieved in 75.8% of patients with severe eosinophilic asthma after 12 months of benralizumab treatment. In addition to the high remission rate, significant improvements were observed in asthma control, annual exacerbation frequency, oral corticosteroid (OCS) use, and lung function parameters. With the increasing use of biologic therapies, clinical remission has emerged as an important therapeutic target in the management of severe asthma [8, 9]. In particular, anti–IL-5 and anti–IL-5 receptor therapies have shown that some patients may achieve a level of disease control extending beyond conventional symptom improvement [10].

Although there is currently no universally accepted definition of clinical remission in asthma, most recent studies define remission as the absence of exacerbations and systemic corticosteroid use together with well-controlled symptoms and stable or improved lung function maintained for at least 12 months [9–11]. The four-component remission definition used in our study was consistent with current consensus recommendations and previously published biologic treatment studies [10, 11]. This multidimensional approach is important because it reflects sustained disease stability rather than symptom control alone.

Reported remission rates with biologic therapies vary considerably according to the remission criteria used, patient characteristics, biologic agent, and duration of follow-up. Recent reviews have reported remission rates ranging from approximately 19% to 43% in real-world studies and from 13% to 35% in post-hoc analyses of randomized controlled trials [12, 13]. However, higher remission rates have also been reported in selected patients with marked type 2 inflammatory features, particularly among those receiving anti–IL-5/IL-5R therapies [14]. The relatively high remission rate observed in our cohort may be related to the prominent eosinophilic phenotype of the study population, the potent eosinophil-depleting effect of benralizumab, and the single-center real-world design of the study. In addition, our study applied a predefined four-component remission definition based on current consensus recommendations, which may differ from the remission criteria used in previous studies. Differences in patient selection, baseline disease severity, and follow-up duration should also be considered when comparing remission rates across studies.

In the exploratory multivariable analysis, lower baseline exacerbation frequency and higher baseline FEV₁ were associated with achieving clinical remission. These findings suggest that patients with a lower disease burden and better preserved lung function at treatment initiation may be more likely to achieve remission following benralizumab therapy. However, given the limited sample size and the small number of patients who did not achieve remission, these findings should be interpreted with caution and considered hypothesis-generating. Larger prospective studies are needed to validate these associations. Recent systematic reviews have shown that higher blood eosinophil counts and FeNO levels may predict better responses to biologic therapies, whereas persistent OCS requirement, poor symptom control, impaired lung function, and comorbidities may reduce the likelihood of remission [14, 15].

Achievement of remission may depend not only on biologic selection but also on the management of accompanying treatable traits. Persistent airway inflammation, airflow limitation, poor treatment adherence, incorrect inhaler technique, smoking exposure, and comorbid conditions may all influence long-term treatment outcomes [16]. Therefore, a multidimensional and individualized treatment approach may increase the likelihood of achieving remission in severe asthma.

Near-complete suppression of peripheral eosinophils following benralizumab treatment was accompanied by marked reductions in exacerbation frequency and significant improvements in lung function. These findings are consistent with both randomized trials and real-world studies demonstrating the effectiveness of benralizumab in severe eosinophilic asthma [3, 4, 7, 17, 18]. The mean increase of 674 mL in FEV₁ observed in our study was clinically notable. In the pivotal SIROCCO and CALIMA trials, improvements in pre-bronchodilator FEV₁ in the benralizumab groups were more modest than those observed in our cohort [3, 4]. Similarly, although real-world studies have consistently demonstrated significant improvements in lung function following benralizumab treatment, the magnitude of FEV₁ gain has varied substantially across cohorts depending on baseline patient characteristics, disease severity, and duration of follow-up [7, 17–19]. The relatively large improvement observed in our cohort may be explained by the lower baseline FEV₁ values, the prominent eosinophilic phenotype of our patients, and the greater potential for functional recovery following eosinophil depletion. Real-world cohorts have also reported reductions in exacerbation rates approaching 90%, and the ANANKE study demonstrated substantial decreases in both overall and severe exacerbations during benralizumab treatment [17]. Long-term follow-up studies have further suggested that these clinical benefits may be sustained over time [7, 19].

The significant reduction in OCS use observed in our cohort is another clinically relevant finding. In the ZONDA trial, benralizumab significantly reduced maintenance OCS requirements, and many patients were able to discontinue OCS completely [20]. Similar reductions in OCS use have also been reported in real-world studies [7]. Current guidelines strongly recommend minimizing long-term OCS exposure because of its well-recognized adverse effects [1]. In addition, the high rate of asthma control achieved in our study indicates that benralizumab may improve not only exacerbation outcomes but also day-to-day symptom control.

The concept of “super-responders” has recently gained increasing attention in the biologic treatment literature. However, super-response and clinical remission are not identical concepts. Clinical remission is generally considered a more stringent and multidimensional treatment target that requires sustained disease control over time [14]. Therefore, the use of predefined objective remission criteria represents an important strength of our study.

Sustained clinical remission may also have implications for future biologic tapering or dosing interval extension strategies. Recent real-world studies have suggested that extending anti–IL-5 biologic dosing intervals may be feasible in selected patients who maintain prolonged four-component remission. However, remission could not be maintained in all patients following a 50% dosing interval extension, indicating that such strategies should be approached cautiously and on an individualized basis [21].

This study has several limitations. First, its retrospective single-center design and relatively small sample size limit the generalizability of the findings. In particular, the limited number of patients who did not achieve clinical remission may have reduced the stability of the multivariable model and increased the risk of model overfitting. Therefore, the results of the multivariable analysis should be considered exploratory and interpreted with caution until confirmed in larger prospective multicenter cohorts. In addition, retrospective real-world data are inherently subject to missing data and clinical heterogeneity. Nevertheless, the study has several important strengths, including the use of predefined multidimensional remission criteria and the evaluation of treatment outcomes under routine clinical practice conditions.

Recent evidence suggests that sustained clinical improvement achieved with biologic therapies may represent not only symptom control but also a potential modification of disease course in selected patients with severe asthma [12].

In conclusion, benralizumab treatment was associated with high clinical remission rates, marked reductions in exacerbations, and significant improvements in lung function in patients with severe eosinophilic asthma. Our findings support benralizumab as an effective biologic capable of achieving clinical remission in selected patients with severe eosinophilic asthma. 

## Data Availability

The datasets generated and/or analyzed during the current study are not publicly available due to institutional and patient privacy restrictions but are available from the corresponding author on reasonable request.
